# A review of virus host factor discovery using CRISPR screening

**DOI:** 10.1128/mbio.03205-23

**Published:** 2024-10-18

**Authors:** Wayne Ren See, Meisam Yousefi, Yaw Shin Ooi

**Affiliations:** 1 Program in Emerging Infectious Diseases, Duke-NUS Medical School, Singapore, Singapore; 2 Infectious Diseases Labs (A*STAR ID Labs), Agency for Science, Technology and Research (A*STAR), Singapore, Singapore; Albert Einstein College of Medicine, Bronx, New York, USA

**Keywords:** virus-host interactions, functional genomics, CRISPR screening, host factors, flavivirus, SARS-CoV-2

## Abstract

The emergence of genome-scale forward genetic screening techniques, such as Haploid Genetic screen and clustered regularly interspaced short palindromic repeats (CRISPR) knockout screen has opened new horizons in our understanding of virus infection biology. CRISPR screening has become a popular tool for the discovery of novel host factors for several viruses due to its specificity and efficiency in genome editing. Here, we review how CRISPR screening has revolutionized our understanding of virus-host interactions from scientific and technological viewpoints. A summary of the published screens conducted thus far to uncover virus host factors is presented, highlighting their experimental design and significant findings. We will outline relevant methods for customizing the CRISPR screening process to answer more specific hypotheses and compile a glossary of conducted CRISPR screens to show their design aspects. Furthermore, using flaviviruses and severe acute respiratory syndrome coronavirus 2 (SARS-CoV-2) as examples, we hope to offer a broad-based perspective on the capabilities of CRISPR screening to serve as a reference point to guide future unbiased discovery of virus host factors.

## INTRODUCTION

All viruses depend on host cell machinery to complete their infection cycle for their survival. The intricate interactions between viruses and their host cells are not one-way processes. Hosts have co-evolved mechanisms to defend themselves against viral infections, for instance, by developing innate immune pathways. On the other hand, viruses have also co-evolved strategies to evade such host innate immune pathways, ultimately resulting in an arms race. For successful replication, viruses must hijack or subvert cellular host factors at all stages of the infection cycle—from entry to viral protein translation, viral genome replication, progeny virion assembly, and exit. Such host factors encompass various cellular components within cells—including host proteins, nucleic acids, and metabolites. Unraveling the identities and functions of these host factors has threefold benefits: first, to fill in the knowledge gaps in the fundamentals of virus infection biology; second, to reveal Achilles heels of clinically important viruses to allow the innovation of antiviral strategies (1); and third, to enhance the repurposing of viruses as gene delivery vectors (2). Occasionally, such research could also have the added benefit of helping to gain insights into the physiological roles of some host factors (3).

Examining random candidate host factors one at a time can be laborious. In recent years, the advances and increased accessibility of various cost-effective technological platforms, from genetic screening, microarrays, transcriptomics, and proteomics to genome-wide association studies (GWAS), have incentivized the field to identify host factors at a larger scale systematically (4–9). The forward genetics approach to dissecting human virus-host interactions at the genome-scale can be divided into gain-of-function or loss-of-function screens. An example of gain-of-function screening is the ectopic expression of cDNA libraries in non-susceptible cell lines, such that an implicated host factor is revealed upon restoration of susceptibility to virus infection (10, 11). On the other hand, early loss-of-function screens relied on chemical or radiation-induced mutagenesis to cause a random loss of function in the genes of genetically tractable model organisms, such as the common fruit fly, *Drosophila melanogaster*, or the nematode worm, *Caenorhabditis elegans*, or even yeast, *Saccharomyces cerevisiae*, but this proved challenging in mammalian cells (12). However, recent loss-of-function screening empowered by various genetic perturbation technologies, such as RNA interference (RNAi), gene-trapping or haploid genetics, and clustered regularly interspaced short palindromic repeats (CRISPR), have successfully fueled multiple unbiased dissections of viral host factors in human cells (12–15).

RNAi targets cellular mRNAs to perturb host cells at the post-transcriptional level (8), while gene trapping introduces permanent mutations to the host genome. Haploid genetic screening utilizes gene-trap constructs to introduce insertional mutagenesis in near-haploid or haploid cell lines, such as HAP1 and eHap. The gene-trap construct carries a splice acceptor site, a fluorescence reporter gene, and a polyA termination signal that is delivered using retrovirus to randomly integrate into the intronic and exonic regions of host cell genomes, perturbing gene expression (16). A pool of these mutagenized cells could then be sorted by flow cytometry and subjected to infection by the virus under study in a live-dead selection. Cells that survive this infection indicate that a gene required for viral infection has been disrupted; thus, mapping the insertional mutagenesis using next-generation sequencing (NGS) will unveil the implicated host factor. As an alternative to the live-dead screening approaches, flow cytometry-based approaches can also be adapted to sort for mutagenized cells exhibiting desired phenotypes—for example, non-infected cells.

Genome-scale CRISPR screening approaches have recently become a popular avenue for genetic studies (14, 17, 18). These screens can generally be categorized based on their approaches, such as the loss-of-function CRISPR knockout (KO) or CRISPR interference (CRISPRi), and the gain-of-function CRISPR activation (CRISPRa). CRISPR KO relies on altering the host genome in a targeted way. The targeting specificity is determined by the single guide RNA (sgRNA), which forms a complex with the Cas9 endonuclease. The sgRNA consists of two parts—the CRISPR RNA (crRNA) region, which is engineered with a sequence complementary to that of the target locus within the genome, and the trans-activating crRNA (tracrRNA) region, which facilitates the binding of the sgRNA to Cas9 endonuclease (19). As such, Cas9 will be guided by the sgRNA to a specific genomic locus, typically to a gene of interest. Cas9 endonuclease catalyzes a double-strand break (DSB) at the specific gene of interest to trigger DNA repair by non-homologous DNA end joining, which can introduce insertions or deletions (indels) that disrupt the gene permanently (20). CRISPRi and CRIPSRa use catalytically dead Cas9 (dCas9) fused with gene expression regulators to drive downregulation or upregulation of gene expression, respectively, without causing any permanent modifications on the genome (21). CRISPRi employs dCas9 fused to a gene repressor, such as KRAB (22), to silence the transcription of a sgRNA-targeted gene (23). In contrast, CRISPRa utilizes dCas9 fused with a transcriptional activator, such as VP64 (22), VPR (a tripartite activator, VP64-p65-RTA) (24), or an activator integrated with additional protein or RNA scaffolds, like SunTag (25, 26) and SAM (27), to upregulate gene expression.

Both gain-of-function and loss-of-function screens have been pivotal in supporting the dissection of virus infection biology. Although each technology has its own unique advantages and disadvantages, no platform is inherently superior. In our experience, for a platform to gain widespread popularity, it must exhibit the qualities of specificity, user-friendliness, reproducibility, and economical (SURE). Most genome-scale genetic perturbation technologies are now commonly used for functional genomics studies due to their increased accessibility and affordability, thus demonstrating their cost-effectiveness. However, while both CRISPR KO and haploid genetics aim to irreversibly knock out genes of interest, CRISPR KO offers the added advantage of specificity as it allows for the targeted KO of a specific gene guided by the sgRNA, whereas haploid genetics depend on random insertions of the gene-trap construct into an intronic region. CRISPRa and ectopic cDNA overexpression both involve a gain-of-function in a gene of interest, but cDNA overexpression libraries can be less robust due to potential biases in gene representation, influenced by mRNA abundance at the time of RNA isolation, unless established and characterized cDNA libraries are used (28). CRISPRi and RNAi both result in downregulation of gene expression without permanent genetic changes, but unlike RNAi, CRISPRi does not compete with endogenous machinery such as microRNA expression or function and thus has been demonstrated to produce a more reproducible and robust knockdown (23, 26). Since CRISPR fulfills the SURE factors, it has become a preferred platform for many studies, which we will focus on in more detail.

**Fig 1:**
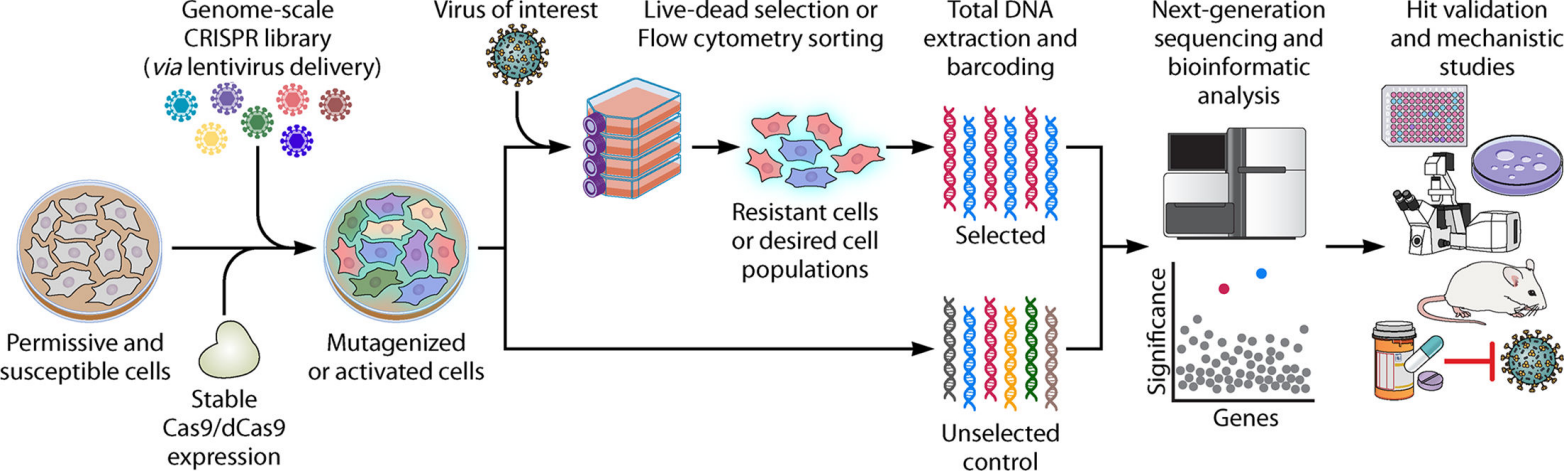
Illustration of the CRISPR screening workflow. Key steps of a CRISPR screening workflow, from selection of suitable cells, the delivery of a pooled sgRNA library, selection of infected cells, to the hit validation after NGS and bioinformatics data analysis.

Just like any molecular virology assays in cell culture, conducting a CRISPR screen to dissect virus-host interactions starts with selecting suitable permissive and susceptible cells. The chosen cells should allow for robust virus replication and proliferation, while obvious cytopathic effects (CPEs) are crucial if a live-dead phenotypic screening approach is opted for. Using a second- or advanced-generation lentivirus delivery system, Cas9 or dCas9 can be introduced into the cell together with the sgRNA in a one-vector system or introduced ahead of the sgRNA in a two-vector system (17, 29, 30). As a result, mutagenized or activated cells will be generated, more commonly in a pool format, although it is also possible to conduct this in an arrayed format (14). Frequently used pooled genome-scale human sgRNA libraries in published screens include the Brunello human genome-wide library (31) or Genome-Scale CRISPR Knock-Out (GeCKO) v2 (30) for CRISPR KO screens and Calabrese (31) or Synergistic Activation Mediator (SAM) pooled library v2 (32) for CRISPRa screens. These libraries are normally designed to target each gene using 3–6 sgRNAs. The pooled mutagenized or activated cells will subsequently be subjected to viral infection for a few days to weeks. Live-dead phenotypic selection or flow cytometric sorting is then utilized to facilitate the identification of implicated host genes following infection with the virus of interest. For a live-dead screen, cells that resist viral infection would survive and proliferate. These resistant cells are likely to carry a specific gene perturbation or an activated gene expression that protects the cell from being infected by the virus. Alternatively, using a fluorescent reporter virus or indirect immunofluorescence staining, non-infected, infected, or highly infected cells can be sorted via flow cytometry. Selected cell populations will then have their total genomic DNA extracted and subsequently prepared for next-generation sequencing (NGS). In the case of Illumina NGS, a common practice is to have the region targeted by the sgRNA amplified and barcoded with Illumina adapters and indexes. The sequencing data acquired from NGS will then be subjected to bioinformatic analyses. Analysis typically involves counting the number of reads for each sgRNA, as sgRNA libraries comprise multiple unique sgRNAs that target different regions of each gene. All genes will be ranked in a hit list based on the overall enrichment or depletion of their sgRNAs. An example of the bioinformatics tools widely used for CRISPR screening analysis is the Model-based Analysis of Genome-wide CRISPR/Cas9 Knockout (MAGeCK) that uses a robust rank aggregation (RRA) algorithm (33). This bioinformatic analysis results in an unbiased discovery of implicated host factors, from which usually top, novel candidate host factors would be validated. Typically, the candidate host genes can be depleted for follow-up assays to ascertain the phenotypic changes in viral infection. To rule out the possibility of off-target effects, cDNA complementation of the KO cells should reverse the observed phenotypes. Given a successful validation, further mechanistic studies can be carried out to better elucidate the role of the validated host factor at a specific stage of the virus infection cycle (Fig. 1).

With the help of CRISPR screening, many new host factors have been discovered and extensively investigated in the context of various biomedically important viruses—from alphaviruses, such as chikungunya virus (CHIKV) (34, 35); human coronaviruses, such as severe acute respiratory syndrome coronavirus 2 (SARS-CoV-2) (36); flaviviruses, such as Dengue virus (DENV) (37, 38); orthomyxoviruses, such as influenza A virus (IAV) (39–41); to retroviruses, such as human immunodeficiency virus (HIV) (42–46). In fact, a few of these studies have gone on to validate their discovered host factors *in vivo,* and after demonstrating a successful protection of viral infection, formed a strong case for host-directed interventions through modulating the host factor discovered from screening (42, 47–53). Considering how many research groups increasingly adopt CRISPR screening for virus-host factor discoveries, this review aims to describe and summarize broad trends observed in recent years. Chiefly, how the CRISPR screening platform has empowered the identification of virus host factors will be exemplified by considering flavivirus and SARS-CoV-2 screens. These examples are chosen for their clinical relevance and, more importantly, can demonstrate how our understanding of viral infection biology is improved based on the wealth of data generated from CRISPR screening. Additionally, from a technical standpoint, a glossary of recent methods will be presented by summarizing how studies have optimized the CRISPR screening process to answer their hypotheses.

## FLAVIVIRUSES

Flaviviruses are enveloped, single-stranded positive-sense RNA (+ssRNA) viruses classified under the *Flaviviridae* family. Owing to their biomedical importance, frequently interrogated viruses within this genus include DENV, West Nile virus (WNV), yellow fever virus (YFV), and Zika virus (ZIKV). Typically, their infection cycle begins upon binding of the viral glycoprotein envelope (E) to an entry receptor or attachment factor on the host cell surface. Clathrin-mediated endocytosis ensues, allowing the virus to internalize into the cell (54, 55). Optimal acidic conditions in the endosome trigger the conformational changes of the viral particle surface E proteins to insert into endosomal membrane, driving membrane fusion. As a result, the viral nucleocapsid is released into the cytoplasm. Upon uncoating, the viral RNA genome recruits the cellular cap-dependent translational machinery to synthesize and insert viral polyproteins into the endoplasmic reticulum (ER) membrane. The polyprotein is then processed by viral and host proteases to form three structural proteins (C, prM, and E) and seven non-structural (NS) proteins (NS1, NS2A, NS2B, NS3, NS4A, NS4B, and NS5). Some of these NS proteins subsequently drive the formation of specialized replication organelles (ROs) by inducing rewiring and curvature of the ER membrane (56). ROs aid in concentrating host and viral factors necessary for viral replication, coordinating the infection cycle, and shielding viral double-stranded RNA replication intermediates from activating RNA sensors and their downstream innate immune responses (57). Newly synthesized viral RNA genomes are exported out of the ROs to interact with capsids (C) to form nucleocapsids. Nucleocapsids will bud into the ER lumen to produce immature progeny virions. During their exit via the cellular protein secretion route, these virions are further processed by host proteases in the trans-Golgi network to become mature infectious particles.

Several cell surface components have been reported to play a role in facilitating flavivirus entry, highlighting the diversity of entry factors that may play redundant roles, and the challenge of pinpointing a specific entry receptor (58). Through CRISPR screens, a few entry factors were identified, such as EXT1 and EXTL3 that are involved in the biosynthesis of glycosaminoglycans, which promote flavivirus attachment to host cells (59). Integrin β5, a component of the integrin αvβ5 was also shown to be required by ZIKV through CRISPR screening (60). It facilitates ZIKV entry into neural stem cells, and blocking integrin αvβ5 with the drug Cilengitide inhibits ZIKV infection. This is corroborated by an investigation into the expression patterns of integrin αvβ5 in the prefrontal cortex, where it was found that while integrin αv expression is proportional to age, integrin β5 is mainly expressed in infants and younger children but not in adults (61). From this, integrin αvβ5 presents as a plausible entry factor, but it should be noted that there have been reports that demonstrate cell line specificity for ZIKV entry (62). For instance, in primary genital epithelial cells, the same amelioration of ZIKV infection could not be observed when integrin αvβ5 was blocked (63). Therefore, integrin αvβ5 might serve as an entry factor for flavivirus infection but only for specific cell types.

Most genome-wide CRISPR KO screens reported for flaviviruses have seen ER-associated host dependency factors being ranked frequently at the top, re-affirming the centrality of the ER in flavivirus infection. Notably, several key components of the N-linked glycosylation pathway, which are responsible for the maturation of glycoproteins and protein folding, were consistently identified and verified as flavivirus host dependency factors. For instance, several subunits of the oligosaccharyltransferase (OST) complex, STT3A (37, 59, 64–66), STT3B (37, 66), OSTC (59, 65), and MAGT1 (37, 66), have been key factors validated in published screens. Flavivirus genome replication was diminished in cells depleted from any of these above-mentioned key subunits of the OST complex. Despite the central role of the OST complex in catalyzing N-linked glycosylation in cells, it was shown that the canonical enzymatic function of the protein complex mediated by the STT3A and STT3B subunits, is dispensable for flavivirus infection (37). Interestingly, the OST complex has been suggested as a potential pan-flavivirus drug target. The pharmacological inhibition of the OST complex using an inhibitor called NGI-1 could potently block the replication of multiple flaviviruses in cells (67). In addition, other ER–protein complexes involved in the N-linked glycosylation process (68), such as the translocon-associated protein (TRAP) and dolichol-phosphate mannose synthase (DPMS) complex, were also identified as critical flavivirus host factors. Key components of TRAP, including SSR2 and SSR3, and subunits of the DPMS Complex—DPM1 and DPM3—have been uncovered by screening to be required for flavivirus RNA genome replication and viral protein glycosylation (69).

In addition to the N-linked glycosylation pathway, factors involved in the ER-associated protein degradation pathway (ERAD), EMC1-6 (59, 64, 65, 70, 71) and SEL1L (65, 71), have been frequently found to be key host dependency factors for flaviviruses. Subsequent investigations have unveiled some of the mechanisms to help explain this phenomenon. The ER membrane complex (EMC), one of the main components of the ERAD, was found to be not only required for the biogenesis of DENV non-structural proteins NS4A and NS4B but also to colocalize with the viral RO through EMC4 and NS4B interaction (72, 73). NS4B would be targeted for degradation in the absence of EMC proteins. Thus, it becomes plausible that one function of the ERAD might be to regulate the turnover of these viral NS proteins. Intriguingly, the EMC may also be involved in other stages of viral infection cycle, such as during membrane fusion. A study demonstrated that the lack of EMC4 could lead to depletion of the phosphatidylserine levels in the late endosome, impacting the endosomal membrane fusion driven by DENV E proteins (74). The ERAD pathway components SEL1L and DERL were similarly proposed to regulate flavivirus NS protein turnover, which is required for efficient viral replication (75). This requirement becomes clear in a study exploring the use of a proteasome inhibitor, Bortezomib, as a potential ZIKV and DENV treatment. They established that two specific E3 ligases are responsible for ubiquitinating viral NS3, which, upon ubiquitination, loses its protease activity required for viral polyprotein self-cleavage (76). When the proteasome is inhibited, an accumulation of ubiquitinated NS3 impairs viral replication. As such, the ERAD can play an important role in regulating viral NS protein levels to allow viral replication to proceed.

TMEM41B and VMP1, two closely related ER-resident proteins that were recently shown to function as phospholipid scramblases that shuffle phospholipids between the ER membrane leaflets (77, 78), have been identified as flavivirus host factors in several CRISPR screens. TMEM41B and VMP1 were shown to be involved in host membrane remodeling and the formation of the ROs that facilitate viral genome replication. Specifically, TMEM41B was described to interact and colocalize with flavivirus NS4A and NS4B at the ER membrane during infection (79). Efficient DENV infection in TMEM41B-deficient cells could be rescued by exogenous fatty acids supplementation—likely through a restoration of ATP production—suggesting that TMEM41B facilitates DENV infection by mediating mitochondrial beta-oxidation (80). Intriguingly, this metabolic rescue did not appear to restore the formation of ROs, while a robust activation of dsRNA sensors, such as RIG-I and MDA5, were triggered, providing new evidence of the importance of ROs in evading the innate immune sensing mechanism (80). Several ER-associated cellular RNA-binding proteins (RBPs) identified from CRISPR screens have been reported to interact with the viral genome to modulate flavivirus genome replication. For example, ribosome-binding RBPs RRBP1 and vigilin (encoded by *HDLBP*) directly interact with flavivirus RNA genome, promoting viral RNA stability and optimal viral translation and genome replication (38). RACK1, a cellular scaffolding protein involved in diverse cellular processes, including signal transduction, was also demonstrated to play a role in viral protein translation prior to the formation of the viral replication complex (81). It is shown that RACK1 binds to the 40S ribosome and vigilin or SERBP1 to interact with the RNA genome, effectively bringing it closer to the translation machinery (82). On the other hand, the ability of RRBP1 to bind viral dsRNA is revealed to be modulated by vimentin, affecting its cellular localization (83).

In addition to ER-resident factors, multiple cytosolic proteins identified via CRISPR screening have been shown to play a role in promoting flavivirus infection. For example, SBDS, a ribosome maturation protein, and SPATA5 (also known as AFG2A), an ATP-dependent chaperone protein, were demonstrated to play role in ribosome biogenesis and viral protein synthesis during YFV infection (84). More recently, PACT (also known as PRKRA), a cellular kinase previously reported to be activated by dsRNA, has been validated as a DENV host dependency factor (85).

The aforementioned studies were CRISPR KO screens, mainly uncovering flavivirus host dependency factors. However, several groups have conducted CRISPRa screens for flaviviruses, unveiling multiple host dependency and restriction factors. Dukhovny et al. (86) identified interferon-stimulated genes (ISGs), like IFN-λ2 and IFI6, as important host restriction factors for ZIKV, helping the host cell to limit virus infection. IFI6 also ranked among top hits in a separate YFV CRISPR KO screen concurrently treated with interferons and was shown to be able to prevent viral RO formation (87). On the other hand, in the CRISPRa screen by Luu et al. (88), RhoV, an atypical GTPase, was validated as an essential host dependency factor proposed to facilitate ZIKV entry.

## SARS-CoV-2

SARS-CoV-2 is an enveloped virus belonging to the *Coronaviridae* family. Each SARS-CoV-2 virion contains a +ssRNA genome, encapsidated by nucleocapsid (N) proteins, and enclosed within a lipid bilayer membrane incorporated with spike (S), envelope (E), and membrane (M) proteins. The key entry event for SARS-CoV-2 involves binding to the cognate proteinaceous receptor, ACE2, via S proteins. Depending on the accessibility of cellular proteases, such as TMPRSS2 and CTSL—to proteolytically cleave S proteins and expose the fusion peptide—SARS-CoV-2 can enter human host cells through membrane fusion at either the plasma membrane or endosomal membrane (89, 90). As a result, the viral genome is released into the cytoplasm. Upon release, the SARS-CoV-2 genome is immediately translated into polyproteins via cap-dependent translation. Subsequently, these polyproteins are processed and matured into multiple individual non-structural proteins (nsp1-16) that regulate viral RNA replication and transcription. They then not only assemble into the SARS-CoV-2 RNA replication and transcription complex (RTC), but also collectively play a role in remodeling the ER-derived membranes to form viral organelles that house the RTC. These viral organelles function as hideouts to protect the replication of the complete viral genome as well as the transcription of sub-genomic viral mRNAs from any interference by the cellular innate immune system. These sub-genomic viral mRNAs encode for SARS-CoV-2 structural proteins (S, E, M, and N) and accessory proteins (ORF3a, ORF3b, ORF6, ORF7a, ORF7b, ORF8, ORF9b, ORF9c, and ORF10). Some of these SARS-CoV-2 accessory proteins are non-essential for viral replication in cell culture but may be involved in viral immune evasion and pathogenesis (91, 92). After exiting the viral replication organelles, full-length viral +ssRNA genomes are packaged by N proteins to form the nucleocapsid, which later bud into cellular compartments containing N, E, and M proteins to form progeny virions. Similar to its entry, SARS-CoV-2 progeny virions have been shown to also be able to exploit several cellular pathways to exit host cells, including the canonical protein secretory pathway and the non-canonical lysosomal exocytosis (93).

Since the beginning of the COVID-19 pandemic, many groups around the globe competed to unbiasedly reveal the infection biology of SARS-CoV-2 using genome-scale CRISPR screening approaches, with a record number of screens being performed on a single virus (36). However, choosing a suitable cell line for CRISPR screening to uncover SARS-CoV-2 host factors proved to be challenging. Viral entry typically requires engaging the cognate receptor ACE2, and subsequently the cell surface protease TMPRSS2 or endosomal CTSL, depending on the entry route. Therefore, suitable cell lines for the genetic screen must express the receptor and at least one or more of these proteases to ensure robust SARS-CoV-2 infection during the phenotypic selection process. For example, cell lines that fulfilled such requirements include VeroE6, an African Green Monkey kidney cell line (94); Calu-3, a human lung adenocarcinoma cell line (95); Caco-2, a human colorectal adenocarcinoma cell line (95); and IGROV-1, a human ovarian cancer cell line (96). For some studies, cell lines, such as A549, a human alveolar adenocarcinoma cell line, and HEK293, a human embryonic kidney cell line, were engineered to ectopically express these entry factors (97, 98).

Through CRISPR screens, more factors facilitating SARS-CoV-2 entry have been revealed in addition to the above-mentioned fundamental entry requirements. Notably, a few studies validated TMEM106B (99, 100), a cellular membrane protein, as a host dependency factor in CRISPR screens utilizing Huh-7, a human hepatoma cell line. A subsequent follow-up paper demonstrated that TMEM106B could act as an alternative SARS-CoV-2 receptor, as supported by evidence of direct interaction between TMEM106B and the SARS-CoV-2 S protein, which facilitated the viral entry in an ACE2-independent mechanism (101). This role of TMEM106B was corroborated in a separate CRISPR screen using HEK293T cells; however, the ACE2-independent entry mechanism was proposed to have limited relevance *in vivo* (102). Additional host factors facilitating the attachment and entry of SARS-CoV-2 have also been identified via genetic screening. Using a Cre‐Gag fusion/flox reporter (103) to screen specifically for entry factors, Hossain et al. (97) identified a component of heparan sulfate biosynthesis, SLC35B2, as a host dependency factor. This is in line with studies that demonstrate the key role of glycosaminoglycans in the entry of SARS-CoV-2, by promoting the binding of the viral spike protein to ACE2 (104–106), as well as its role in spike-induced cell-cell fusion (107).

Since some CRISPR screens were designed to favor the entry of SARS-CoV-2 into host cells via clathrin-mediated endocytosis, it was reasonable to observe multiple components of endocytic pathway appearing as top hits. For instance, AP1G1, a subunit of clathrin-associated adaptor protein complex 1 (50, 95, 108), and components of the phosphoinositide 3-kinase (PI3K) pathway that plays a role in vesicular trafficking (109)—such as PIK3C3, a catalytic subunit of the PI3K complex (99, 110); WDR81 and WDR91, negative regulators of PI3K activity (98, 111, 112)—were all validated as hits in several screens. RAB7A, a GTPase that facilitates the formation of the retromer complex, was also found to be a host dependency factor in CRISPR screens (98, 110, 113–115). It was shown that RAB7A deficiency would sequester ACE2 in endosomes; hence, playing a key role in regulating ACE2 expression on the cell surface (110). Additionally, CCZ1, a guanine exchange factor for RAB7A, was separately validated in other screens (98, 116). Components of the Commander (CCC) complex was also discovered to be required for SARS-CoV-2 endocytosis. Multiple CCC complex subunits, such as CCDC22, CCDC93, CCDC53, and COMMD2-10, were validated and may play a role in the endosomal transport of the virus (98, 110, 111, 117). This observation is consistent with the known role of the CCC complex in endosomal cargo trafficking, as described previously (118, 119). Likewise, the retromer complex, which consists of vacuolar protein sorting (VPS) sub-complex and one or more sorting nexins, was found to play a critical role in SARS-CoV-2 infection. All subunits of VPS, VPS26, VPS29, and VPS35, were verified as SARS-CoV-2 host dependency factors across several CRISPR screens (98, 110, 111, 117). The retromer complex is known to be involved in the sorting and trafficking of transmembrane proteins from endosomes to the trans-Golgi network and the cell surface (120). However, a report demonstrated that a specific retromer complex containing the sorting nexin SNX27 was able to sort internalized SARS-CoV-2 virions to the recycling pathway at the early endosome, thereby preventing the virus from reaching and fusing into the late endosome (121). PLAC8, a poorly studied cellular protein, is another host factor suggested to affect endosomal entry of SARS-CoV-2 through mediating the autophagolysosomal compartment (117). PLAC8 is highly expressed in both ciliated cells of the respiratory tract and in gut enterocytes, and post-mortem analysis of COVID-19 patients has demonstrated its requirement for successful SARS-CoV-2 infection in the pancreas (122). PLAC8 was also identified as a host dependency factor for swine acute diarrhea syndrome coronavirus (SADS-CoV), another member of *Coronaviridae* (123).

Upon entering host cells, SARS-CoV-2 must hijack and rewire cellular metabolism to fuel its translation, replication, transcription, and exit (91). Some cellular factors that regulate cellular metabolism, such as SCAP (100, 114) and SREBF2 (also known as SREBP2) (113, 114), have been identified to be critical for SARS-CoV-2 infection. SCAP is an ER chaperone for SREBF1 and SREBF2 proteins, which are transcription factors that play a major role in cholesterol synthesis and homeostasis (124). SARS-CoV-2 uses SREBF2 to upregulate lipid production for replication, resulting in the activation of pyroptosis that induces inflammatory cytokine production (125). Similar to flaviviruses, TMEM41B also plays a multifaceted role during SARS-CoV-2 infection and is identified across several genetic screens (99, 113, 126, 127). Since TMEM41B is known to modulate lipid metabolism (126), it is likely to play a role in a post-entry step of SARS-CoV-2 infection (99). TMEM41B has been demonstrated to mediate the formation of the DMVs at the ER, which facilitates SARS-CoV-2 viral replication (128). TMEM41B was also found in a CRISPR screen for transmissible gastroenteritis virus (TGEV), a porcine coronavirus from the *Coronaviridae* family, and was shown to assist virus internalization in addition to its role in formation of DMVs (129). Other cellular factors that are involved in lipid and cholesterol metabolism—such as adipocyte lysoplasmalogenase TMEM86A (130, 131) and intracellular cholesterol transporters NPC1 and NPC2 (98, 110, 114)—were validated to play a role in promoting SARS-CoV-2 infection. SLC7A11, which forms a heterodimeric amino acid transporter with SLC3A2 to mediate the exchange of extracellular anionic L-cystine and intracellular L-glutamate across the plasma membrane, was validated and demonstrated to facilitate viral-induced reactive oxygen species (ROS) accumulation, which subsequently promotes viral replication (132–134).

Various host dependency factors involved in epigenetic regulation have been described to be important for SARS-CoV-2 infection. The histone acetyltransferase EP300 (95, 108, 113) is one such example identified from CRISPR KO screening. The chromatin remodeler was suggested to work in conjunction with the histone demethylase, KDM6A, and the histone methyltransferase, KMT2D, on the enhancer regions of ACE2 to promote viral entry (135). Consistently, other published screens also validated the related histone methyltransferase, KMT2C, as a host dependency factor (95, 136). Additionally, HMGB1, the non-histone nuclear DNA-binding protein; SMARCA4, a component of the SWI/SNF chromatin remodeling complex; and DYRK1A, a dual-specificity tyrosine phosphorylation-regulated kinase were identified in a CRISPR screen using African green monkey VeroE6 cells (94). HMGB1 was demonstrated to facilitate chromatin accessibility at the ACE2 locus, thereby promoting ACE2 expression on the cell surface (94). SMARCA4 similarly promotes chromatin accessibility at the ACE2 locus, but through the SWI/SNF complex (137). DYRK1A was confirmed to promote DNA accessibility at the ACE2 promoter and enhancer regions, similarly promoting ACE2 expression and facilitating SARS-CoV-2 infection (138). In a separate screen, histone lysine methyltransferases EHMT1 and EHMT2, alongside the E3 ubiquitin ligase TRIM33 were proposed to facilitate viral replication, whereas another E3 ubiquitin ligase, TRIM28, was suggested to facilitate viral exit (139). The transcription factor MAFG, a Maf family protein that complexes with DNA methyltransferases and chromatin remodelers to effect epigenetic regulation, was also identified as a host dependency factor for SARS-CoV-2 (130, 140). AHR, a ligand-activated cytosolic transcription factor involved in the regulation of biological responses to planar aromatic hydrocarbons, was found to be a host dependency factor in a comparative CRISPR screen performed between the betacoronaviruses SARS-CoV-2 and human coronavirus OC43 (HCoV-OC43) (96). It was demonstrated that AHR dampens the type I interferon (IFN-I) response in cells and upregulates ACE2 transcription to promote SARS-CoV-2 infection (141). AHR signaling has also been shown to be important for SARS-CoV-2 infection in patients, which was also corroborated by pharmacological inhibition *in vivo* (142–144).

On the other hand, CRISPR screens have also identified pertinent host restriction factors that play a role in restricting SARS-CoV-2 infection. For example, mucins—MUC1 and MUC4—were found to be able to inhibit SARS-CoV-2 replication and were validated in a murine model (50). Several mucin proteins were subsequently demonstrated in a separate study to play a protective role in inhibiting SARS-CoV-2 infection at the human respiratory epithelial surface, presumably by preventing the virus from engaging ACE2 (145). Another CRISPR screen discovered LRRC15, a plasma membrane protein, as a SARS-CoV-2-binding protein that could sequester the virions and restrict infection (146). Other independent studies corroborated that LRRC15 is able to bind the S protein and block SARS-CoV-2 entry (147, 148). OAS1, a well-known oligoadenylate synthase that senses viral dsRNA and activates the RNase L-mediated degradation of viral RNA (149), was found to protect host cells against SARS-CoV-2 infection (150). Data from COVID-19 patient samples corroborate this as OAS1 is upregulated during COVID-19 infection, and its deficiency exacerbates the disease (151, 152). Besides activating RNase L, OAS1 also exerts antiviral activity upon prenylation by inhibiting SARS-CoV-2 DMV formation (153). DAXX, a multifunctional transcription co-repressor, has been identified in an ISG-focused screen as a restriction factor inhibiting SARS-CoV-2 infection post-entry (154). Interestingly, the study had found that SARS-CoV-2 has evolved a strategy to counteract DAXX via nsp3-induced proteosomal degradation of the host restriction factor. CRISPR screening approaches have also been proven to be useful as evidenced by the identification of some previously discovered host restriction factors. For instance, LY6E, a known restriction factor for multiple coronaviruses, including MERS-CoV, SARS-CoV, and HCoV-OC43 (155, 156), was also identified as a hit in SARS-CoV-2 CRISPR screens (150, 154). LY6E is a GPI-anchored cell surface protein that acts as a toll to restrict SARS-CoV-2 entry. A more recent *in vivo* study demonstrated that LY6E is a key host restriction factor in limiting coronavirus infection in the respiratory tract of mice, and its abrogation increased viral burden and worsens infection-associated pathology (157).

In sum, CRISPR screening technologies have expedited unbiased discoveries of many essential host factors for flaviviruses and SARS-CoV-2. However, accurately defining a critical subset of host factors for each virus species remains difficult, and there remain significant gaps in formulating a comprehensive understanding of the role each host factor plays during viral infection. To aid further inquiry into the host factors that have been discussed, those that have been validated from flavivirus and SARS-CoV-2 screens have been summarized in Table 1.

**TABLE 1:** Summary of CRISPR screens conducted in SARS-CoV-2 and flaviviruses^*a*^

Virus species	Virus strain	Type of CRISPR screen	sgRNA library	Cell line	Validated host factors	Citation
SARS-CoV-2	AF488-conjugated Spike protein	a	Calabrese P65-HSF	HEK-293T	ACE2, LLRC15	(146)
	BavPat1	KO	Brunello	Calu-3	ACE2, AP1G1, CTDSPL2, CUL5, DUSP7, EFHB, EP300, EXTL2, GATA6, IRF6, PRORY, PRR18, RLN1, SLC35F4	(108)
	GHB-03021	KO	Brunello	Huh-7	EXT1, OSBPL9, PIK3C3, TMEM106B, TMEM30A, TMEM41B	(99)
	H-Ap20-1	KO	GeCKO v2	Calu1-ACE2	ACE2, CCDC53, COMMD2, CTSL, PLAC8, SPNS1, VPS26A, VPS29	(117)
	IDF0372	KO	Brunello	Caco-2	AAGAB, ACE2, AP1B1, AP1G1, ATP8B1, EP300, KMT2C, TMPRSS2	(95)
			*C. sabaeus*	Vero E6		
			Gattinara	Calu-3		
	IDF0372	a	Calabrese	Calu-3	ATAD3B, ATP4V0A2, BHLHA15, CD44, CUX1, FBXL19, FXYD5, IL6R, IFNL2, LY6E, LYN, MAFK, MUC1, MUC21, MUC4, PLAGL1, TEAD3, ZNF572	(95)
	IDF0372	KO	Human ISG CRISPR KO	A549-ACE2	CTSL, DAXX, LY6E	(154)
	nCoV-SH01	KO	Brunello	A549-ACE2	ACE2, ACTR2, ACTR3, ARPC3, ARPC4, ASCC3, C16ORF62, C18ORF8, CCDC22, CCDC53, CCDC93, CCZ1, CCZ1B, COMMD10, COMMD2, COMMD3, COMMD4, COMMD5, COMMD7, COMMD8, CTSL, KIAA0196, KIAA1033, NPC1, NPC2, RAB7A, TFE3, VPS29, VPS35, WDR81, WDR91	(98)
	SB3-TYAGNC	KO	Toronto Knockout v3	Vero E6, Calu-3, UM-UC-4, HEK-293, Huh-7	ACE2, KMT2C, TMPRSS2	(136)
	Singapore/2	KO	Brunello	IGROV-1	AHR	(96)
	UF-1	KO	Brunello	HEK-293T-ACE2	CCZ1, CTSL, EDC4, XRN1	(116)
			*C. sabaeus*	Vero E6		
	USA-WA1	KO	GeCKO v2	A549-ACE2	ACE2, ATP6AP1, ATP6V1A, BAX, CCDC22, CNOT4, COMMD3, CTSL, CXCL5, FBXO27, FBXO33, HDCA9, HNRNPC, INO80E, NPC1, PCID2, PIK3C3, PRKCA, PSMB5, RAB7A, SIGMAR1, SNX27, SPEN, TMEM165, TMPRSS2, TOR1AIP1, TRIM4, VPS35, ZC3H18	(110)
	USA-WA1	KO	*C. sabaeus*	Vero E6	ACE2, ARID1A, ATRX, CABIN1, CTSL, DOLK, DPF2, DYRK1A, HIRA, KDM6A, PCBD1, PHF6, PIAS1, PIAS2, RAD54L2, SMARCA4, SMARCA5, SMARCE1, UBXN7	(94)
	USA-WA1	KO	Brunello	Huh-7.5	ACE2, BMPR1A, CDX2, EP300, NRIP1, RAB7A, SREBF2, TMEM41B, VMP1	(113)
	USA-WA1	KO	GeCKO v2	Huh-7.5.1	ACE2, EXOC2, MBTPS2, SCAP, TMEM106B, VAC14	(100)
	USA-WA1	KO	Custom	Huh-7.5	ACE2, EMC1, GPAA1, HS2ST1, INSIG1, NPC2, PIGO, PIGS, RAB10, RAB14, RAB18, RAB1A, RAB5C, RAB7A, SCAP, SREBF1, SREBF2, VPS11, VPS39	(114)
	USA-WA1	KO	Brunello	Calu-3	ACE2, AP1G1, CHUK, RIPK4, ROCK1, TAOK2, TMPRSS2	(50)
	USA-WA1	a	Calabrese	Calu-3	CCNE1, CD44, JPD2, MUC1, MUC13, MUC21, MUC4, SPDEF, TEAD3, ZNF275	(50)
	USA-WA1	a	Custom: ISG	A549-ACE2	CD74, CTSS, ERLIN1, LY6E, OAS1, TRIM25	(150)
	USA-WA1	KO	Custom	Definitive Endoderm cells	ACE2, CTSV, FOXO1, L3MBTL4, MAFG, TCEA3, TMEM86A	(130)
	USA-WA1	KO	Custom	Calu-3	IFNAR1	(158)
	Pseudotyped USA-WA1	KO	Brunello	HEK-293T-ACE2	ACE2, SLC35B2	(97)
	CLIMVIB2, MIG457, USA-WA1	KO	GeCKO v2	Calu-3	ACE2, ATP13A2, CMC4, CNPY3, ECHDC3, EEPD1, MASTL, METTL15, RIPK4, SAR1A, SLC7A11, STAT2, TMCC1, TMPRSS2, TMPRSS4, TTC31	(132)
	UT-NCGM02	KO	Human Improved Genome-wide KO	A549	EHMT1, EHMT2, TRIM28, TRIM33	(139)
Flaviviruses						
DENV	DENV2-16681	KO	GeCKO v2	Huh-7.5.1	MAGT1, STT3A, STT3B	(37)
DENV	DENV2-16681	KO	GeCKO v2	Huh-7	MAGT1, STT3A, STT3B	(66)
DENV	DENV2-16681	KO	Custom	Huh-7	PACT	(85)
DENV	DENV1-276RKI	KO	GeCKO v2	Huh-7.5.1	HDLBP, RRBP1	(38)
	DENV2-429557					
	DENV3-H871856					
	DENV4-BC287/97					
DENV	DENV2-JAM	KO	GeCKO v2	HAP1	DPM1, DPM3	(69)
JEV	RP9	KO	PigGeCKO	PK-15 (Porcine)	B3GAT3, CALR, EMC3, GLCE, HS6ST1, SLC35B2	(159)
WNV	NY99	KO	Custom	HEK-293T	DERL2, EMC2, EMC3, HRD1, SEL1L, UBE2G2, UBE2J1	(71)
WNV	New York 2000	KO	GeCKO v2	HEK-293T	EMC4, EMC6, HSPA13, OST4, OSTC, SEC61B, SEC63, SEL1L, SERP1, SPCS1, SPCS3, STT3A	(65)
YFV	17D	KO	Brunello	Huh-7.5 +IFN I treatment	IFI6	(87)
YFV	17D	KO	Brunello	Huh-7.5	SBDS, SPATA5	(84)
YFV	Asibi	KO	GeCKO	B3GALT6^-/-^ HAP1	TMEM41A, TMEM41B, TMEM64, VMP1	(79)
ZIKV	PRVABC59	KO	GeCKO	B3GALT6^-/-^ HAP1	TMEM41A, TMEM41B, TMEM64, VMP1	(79)
ZIKV	PRVABC59	a	SAM v1	STAT1^-/-^Fibroblasts	RhoV, WWTR1	(88)
ZIKV	MR766	KO	Brunello	Neural Progenitor Cells	ATP6AP2, ATPV1B2, EMC1, EMC2, EXT1, EXT2, EXTL3, OSTC, SLC35B2, SND1, STT3A, TMEM165, TMEM41B	(59)
ZIKV	MR766	KO	GeCKO v2	H1-HeLa	AXL, EMC1, EMC2, EMC3, EMC4, EMC6, NIPAL2, STARD10, STT3A, WDR7, ZFYVE20	(64)
ZIKV	MR766	KO	Human Activity-Optimized CRISPR KO	Neural Progenitors	ATP6V1C1, ATP6V1F, C3ORF58, EMC2, EMC6, ISG15, SOCS3, SSR2, SSR3, STAT3, TM9SF2	(70)
ZIKV	MR766	KO	GeCKO v2	Huh-7.5	EMC1, EMC6, RACK1	(81)
ZIKV	MR766	a	SAM v2	Huh-7	IFI6, IFN-λ2	(86)
ZIKV	ZsGreen	KO	Brunello	Glioblastoma stem cells	BAALC, CENPH, EPHA10, GCNT7, HOMER1, ITGB5, MYLPF, PTPN2, TRAM1	(60)

^*a*^ a = activation; KO = knockout.

## CUSTOMIZED SCREENING APPROACHES

Beyond mechanistic insights, there remain noteworthy lessons that can be learnt from the recent developments in genome-scale CRISPR screening methodologies. Conventionally, most of the published studies often make use of established human transformed or cancer cell lines, and as discussed, have been largely successful in unveiling essential virus host factors in a robust and cost-effective manner. However, in hopes of addressing more specific hypotheses, recent studies have employed strategies to streamline the screening process. As an example, instead of targeting the entire host genome, smaller-scale sgRNA libraries can be helpful to address certain specific questions (85, 114, 160, 161), such as customized CRISPR libraries targeting ISGs (150, 154, 162, 163).

Engineering cells present as a promising avenue to optimize CRISPR screening. Often, this is done to increase the susceptibility of cells to viral infection. For instance, Orchard et al*.* (164) used HeLa cells (HeLa-CD300lf) engineered to be susceptible to murine norovirus (MNoV) to reveal human TRIM7 as a host restriction factor. More recently, a group had conducted a screen in a HT-29 cell line that is deficient in common attachment factors, such as heparan sulfate proteoglycans (HSPGs) and sialic acids for human parechovirus (PeV-A) infection (165). Considering that such attachment factors are hijacked by various viruses for attachment to host cells (166), the group had depleted the expression of HSPGs in their screening so that the identification of cognate protein receptors would be favored. Cells have also been engineered to enable FACS selection of heavily infected or non-infected cells. A study had expressed the herpes simplex thymidine kinase fused to a green fluorescent protein in HeLa cells (HeLa-tkGFP) to provide visual representation of cell death and revealed ganglioside involvement in hepatitis A virus (HAV) infection (167). Another group engineered GXRCas9—a CD4+ T cell line overexpressing CCR5, Cas9, and GFP—to drive physiological relevance and enable FACS selection for their screen in HIV infection (45). An example of FACS selection following immunofluorescence staining is through the use of HepG2 cells containing an integrated 1.1-mer hepatitis B viral genome (HepG2-HBV) (168). The study was interested in factors regulating transcription of HBV surface antigen (HBsAg) in chronic HBV, thus this engineered cell line allowed FACS-based selection of such cells following HBsAg staining. In addition to genetic modifications of cellular systems, it is also possible for screens to use chemical treatment, for instance, to prime the activation of a specific innate immune pathways that are normally inactive. Some CRISPR KO screens have successfully used IFN-I pre-treatment to identify host factors that play a role in antiviral defense (87, 169, 170). Similarly, treatment of cells with antiviral drugs to characterize its unknown mechanism of action can also be done to uncover its cellular targets or even understand host factors that drive drug resistance with CRISPR screening (171, 172).

Notably, a few studies managed to conduct screens in primary cells and stem cells. Itell et al. (162) revealed novel ISGs involved in HIV infection in their CRISPR KO screen on primary human CD4+ T cells. Pertinent examples of human stem cell usage that have been previously discussed are neural progenitor cells (59, 70) and glial stem cells (60) in flaviviruses, as well as the haploid human embryonic stem cell-derived definitive endoderm (DE) cells in SARS-CoV-2 (130). Besides this, we anticipate organoid systems becoming a popular platform for CRISPR screening of virushost interactions as they are more physiologically relevant, given their recent successes in studying cancer and neurological diseases (173–175).

Beyond human cells, the use of non-human cells has also been made possible. Murine cell lines top this list given their extensive usage as model organisms and their well-studied genome. Zhang et al. (35), for instance, utilized 3T3 cells to conduct a CRISPR KO screen with multiple arthritogenic alphaviruses and validated MXRA8 as a receptor for these viruses in human cells. Murine cells like BV-2 have also been used to understand rodent-borne viruses, like MNoV (176). In the case of a murine cytomegalovirus (MCMV) CRISPR KO screen, 3T3 cells had to first be engineered to abrogate Nrp1 expression to allow the researchers to demonstrate the requirement of β-2-microglobulin and Cd81 for MCMV infection of murine macrophages (177). In like manner, Ma et al. (178) experimented on B4galt7-deficient mice Neuro2a cells to screen for entry factors for Venezuelan equine encephalitis virus (VEEV). Consequently, with the use of non-human cell lines, the sgRNA library used must also be tailored to the species in order to successfully generate the genome-wide KOs. In the case of mice, the use of the Genome-wide Mouse Lentiviral CRISPR gRNA Library v1 (179), its later improved version (180), or the Brie library (181) is common. Furthermore, Snyder et al. (182) engineered C57/BL6-derived embryonic fibroblasts to investigate reovirus infection using a CRISPR KO screen. Murine embryonic stem cells have similarly been used in a CRISPR KO screen for murine leukemia virus (MLV) (183). Several groups have used porcine cell lines in CRISPR screens to elucidate host factors for viruses that are well-known to infect pigs, for example, the flavivirus Japanese encephalitis virus (JEV) (159), porcine deltacoronavirus (PD-CoV) (184), and porcine reproductive and respiratory syndrome virus (PRRSV) (185). Notably, the CRISPR KO screen for JEV also uncovered a subset of ER-associated host factors with similar human orthologs that are required for other flaviviruses to infect human cells (159). Additionally, the wild boar lung cell line, WSL-R-HP, was also used in a screen investigating African swine fever virus (ASFV) infection (186). Compared with mice, such porcine studies are not as common, as the pig genome has not been extensively studied. Thus, these studies have to design their own CRISPR sgRNA libraries like PigGeCKO (159) or SsCRISPRko.v1 (187). Another great example of non-human CRISPR libraries are the *Chlorocebus sabaeus* sgRNA libraries, which were engineered to screen for host factors for SARS-CoV-2 (94), and porcine epidemic diarrhea virus (PEDV) (188) in African green monkey cell lines, such as VeroE6 cells. Interestingly, a recent study indicated that it is possible to perform CRISPR screens in a bat cell line, such as the *Pteropus alecto* to interrogate influenza A virus (IAV) host factors (189). Given that such bats are the natural reservoirs of potential zoonoses, this presents a viable opportunity to drive investigations into emerging zoonotic viruses with pandemic potential.

Apart from engineering cells, viruses can also be engineered to execute CRISPR screens. For instance, lentiviral constructs and the full-length HIV genome were integrated with CRISPR/Cas9 libraries that targeted selected subsets of ISGs to allow the modified virus to direct the screening of host factors that limit viral replication and spread (169, 190). In another innovative effort to screen for novel IAV host dependency factors, the IAV genome was integrated with a modified CRISPR/dCas9 system to direct a CRISPRa screen in human cells (191). Coining the technique as transcriptional regulation by pathogen-programmed Cas9 (TRPPC), a cytoplasmic DNA exonuclease, TREX1, was validated as a host factor that dampens innate immune responses to promote IAV infection. Similarly, a recent study had also successfully integrated sgRNA libraries into the HCMV genome, to conduct an incipient virus-encoded CRISPR-based direct readouts screening (VECOS), which allowed them to identify and profile host factors at different stages of viral infection (192). In another approach to exclude HSPG hits to screen for host factors involved in post-entry pathways in IAV, a group had modified the virus itself, pseudotyping IAV with VSV-G to bypass glycan-mediated entry pathways and found IFIT2, an ISG, that promotes viral gene expression (193). Instead of requiring higher biosafety containment facilities, i.e*.*, BSL-3 and BSL-4 labs, pseudotyped or attenuated viruses present a safer alternative to study viruses at the BSL-2 environment (178). A recent CRISPR KO screen using Ebola virus (EBOV) lacking VP30, a viral protein required for viral transcription, had successfully validated SLC39A9 and PIK3C3 as key entry factors for EBOV, at BSL-2 (194). By ectopically expressing VP30 in selected cell lines, successful infection was only possible in these engineered cells, thus demonstrating how virus and cell line engineering can provide biological containment for studying highly pathogenic viruses.

Beyond cell and virus engineering, employing various creative CRISPR/Cas perturbation methods can also be advantageous. In addition to Cas9, the application of other Cas proteins, such as Cas12a and Cas13, could be potentially rewarding. For example, Cas13 empowers CRISPR editing on RNA transcripts, providing an alternative to CRISPRi for post-transcriptional gene regulation (195). Instead of depleting one gene in each cell, multiple gene KOs can be achieved with advanced sgRNA expression vectors (14). For instance, a recently reported CRISPR/Cas12a multiplex genetic perturbation allowed screening for complementary or redundant host factors along the same signaling pathway (196). Additionally, improved CRISPR/Cas delivery methods like self-deliverable ribonucleoproteins or peptide-assisted genome editing (197, 198) could circumvent issues associated with lentiviral or retroviral transduction, including triggering unwanted cellular innate immune responses or random integration of DNA into genome. Advancements in CRISPR/Cas9 delivery systems may enable the perturbation of the mitochondrial genome, facilitating the screening of mitochondrial genes (199). Newer engineered Cas systems like CRISPR Prime editing, which can incorporate short sequences of DNA into the genome using an engineered reverse transcriptase and a prime editing gRNA (pegRNA), allow for pooled screening of genetic variants (200, 201). We believe that this new technology holds potential in follow-up studies to comprehensively dissect virus-host interactions at the nucleotide level and discover critical amino acid variations in cellular factors important for viral infection. Additionally, current CRISPR KO or CRISPRa screening platforms can also be integrated with proteomics (38), metabolomics (202, 203), glycomics (204, 205), and single-cell spatial transcriptomics (206, 207) technologies to enhance the functional insights into virus-host interactions (208).

All CRISPR screening outcomes are ultimately revealed using next-generation sequencing; therefore, the analysis of NGS data is a crucial step in determining hits. In terms of analysis of NGS data, MAGeCK is widely used for its user-friendliness and established reliability. Other bioinformatics algorithms like HiTSelect (209) and STARS (181) are also occasionally used. Additionally, some studies have also used their own customized analysis methodologies, generally encompassing various statistical tests or specific ranking criteria to refine their hit list.

Despite all these variations between screens, studies have the common objective of identifying host factors that are implicated in the infection cycle of the virus in question. Yet, a comparison of the top hits between studies performed on the same, or similar viruses, presents a conundrum. In most cases, there is almost always no consensus between screens. What one screen considered to be the top hit is not necessarily found in other screens for the same virus. This is likely due to the technical differences between screens, for instance the cell lines used typically differ. Hence, it becomes difficult to compare hits across screens. Ultimately, the answer to this lies in the ability to compare data between screens. Meta-analysis should enable one to integrate hit lists from various screens. A benefit of doing so is to allow common or unique host factors across multiple screening results to be revealed for further investigation. Oftentimes, studies do not pursue further analysis of their hits beyond the initial ranking. It might be possible that there remain host factors that might not appear significant when analyzed within the context of the individual screen but when compared across screens, prove to be involved.

To exemplify this idea, MaGplotR is a tool that was recently developed to help visualize comparisons across CRISPR screen data sets (210). The purpose of the tool is to aid in the analysis of screens that generate multiple data sets, often because of either biological replicates or different screening conditions. A drawback is that it can only take hit lists analyzed by MAGeCK as its input, thus limiting users from integrating results obtained through other analysis methods. Another available tool for the meta-analysis of screening data is Meta-Analysis by Information Content (MAIC), which is designed to be able to integrate ranked and un-ranked gene lists, including results from CRISPR screens and data from annotated pathways, RNAi screens, and -omics data (41). This platform has been used in another study to identify lesser-known host pathways that could be of interest in IAV infection (211). More recently, Gene Rank Meta aNalyzer (GeneRaMeN) has been developed to facilitate the meta-analysis of hit lists across various genetic screens. In addition to identifying genes that are commonly found across the input hit lists, called rank aggregation, GeneRaMeN can also detect genes that ranked highly in only a subset of screens, named Rank Uniqueness, and identifies gene sets exhibiting similar or opposite ranking trends across different data sets, termed rank correlation (212). By relying on the hit ranks instead of the enrichment scores, GeneRaMeN eliminated the need to normalize scores across studies, thus enabling the users to incorporate screens analyzed via various pipelines, including MAGeCK and custom methodologies. Through integrating haploid and CRISPR screens for flavivirus host factors, the authors demonstrated that the set of flavivirus host factors identified in each study is significantly impacted by the choice of cell line, emphasizing the importance of meta-analyzing genetic screen results to obtain a more comprehensive insight. Such meta-analyses also facilitate the consolidation of redundant biological pathways within a single hit list, as exemplified by the concurrent identification of both TMPRSS2 and CTSL proteases in SARS-CoV-2 CRISPR screens using GeneRaMeN’s Rank Aggregation.

Hence, these tools have illustrated the utility of meta-analysis in CRISPR screens, which can be helpful in furthering our understanding of infection biology. It is conceivable that with the development of more advanced meta-analysis methodologies, more and more insights can be extracted from already published screens, beyond those initially reported. To conclude, CRISPR screening has been useful in uncovering both host dependency and restriction factors as illustrated in the flavivirus and SARS-CoV-2 case studies. Currently, many groups have started customizing their approach for their CRISPR screens—utilizing primary cells, stem cells, engineered cells, non-human cells, or even cell lines derived from exotic animals like pigs or bats. This is done all in a bid to refine their screening results to obtain a more specific list of host factors that can better answer the hypothesis at hand. In line with this, meta-analysis can be a way to integrate results from various screening approaches to possibly provide a novel perspective on the hits from published data sets.

Beyond flavivirus and SARS-CoV-2 screens, an extensive list of studies has been mentioned in turn to highlight some of the innovations adopted to better understand infection biology of several human and animal viruses. In Table 2, these studies have been outlined alongside other pertinent studies that have explored various techniques in their CRISPR screens. By no means is this table exhaustive, and the goal here is to provide a repository to provide one with a reference point for their own CRISPR screen, should one be considering design aspects.

**TABLE 2:** Glossary of various strategies CRISPR screens have adopted^*a*^

Virus family	Virus species	Virus strain/serotype	Type of CRISPR screen	sgRNA library	Cell line	Algorithm	Selection	Citation
*Arenaviridae*	LCMV	ARM-4	KO	GeCKO v2	A549	MAGeCK	FACS	(213)
*Arteriviridae*	SHFV	LVR	KO	*C. sabaeus*	MA-104	MAGeCK	Survival	(214)
	PRRSV	VR-2332	KO	*C. sabaeus*	MA-104	MAGeCK	Survival	(214)
	PRRSV	WUH3	KO	Custom	PK15 (porcine)	Custom	Survival	(185)
*Asfaviridae*	ASFV	ad-7GD	KO	Custom	WSL (boar)	MAGeCK	Survival	(215)
	ASFV	Armenia	KO	SsCRISPRko.v1	WSL-R-HP (boar)	MAGeCK	Survival	(186)
*Caliciviridae*	MNoV	CR6, CW3	a	Calabrese	HeLa-CD300lf	STARS	Survival	(164)
	MNoV	CR6, CW3	KO	Asiago	BV-2	STARS	Survival	(216)
	MNoV	MNV-S7	KO	Custom	RAW 264.7 (murine)	Custom	Survival	(176)
*Coronaviridae*	HCoV-OC43	VR-759	KO	Brunello	A549	MAGeCK	Survival	(111)
	HCoV-229E	VR-740	KO	GeCKO v2	Huh-7	MAGeCK	Survival	(126)
	HCoV-229E	VR-740	a	Calabrese	Huh-7	MAGeCK	Survival	(217)
	HCoV-229E	N/A	KO	GeCKO v2	Huh-7	MAGeCK	Survival	(127)
	MERS-CoV	EMC	KO	GeCKO v2	Huh-7	MAGeCK	Survival	(127)
	PDCoV	CHN-HN-2014	KO	Custom	LLC-PK1 (porcine)	Custom	Survival	(184)
	PDCoV	CHN-SC2015	KO	Custom	LLC-PK (porcine)	MAGeCK	Survival	(218)
	PEDV	JS-A	KO	VeroCKO	Vero E6	Custom	Survival	(188)
	SADS-CoV	N/A	KO	Brunello	Huh-7.5	MAGeCK	Survival	(123)
	SARS-CoV-2	AF488-conjugated Spike protein	a	Calabrese P65-HSF	HEK-293T-ACE2	MAGeCK	FACS	(146)
	SARS-CoV-2	BavPat1	KO	Brunello	Calu-3	MAGeCK	Survival	(108)
	SARS-CoV-2	H-Ap20-1	KO	GeCKO v2	Calu1-ACE2	MAGeCK	Survival	(117)
	SARS-CoV-2	IDF0372	KO	Brunello	Caco-2	Custom	Survival	(95)
	SARS-CoV-2	IDF0372	KO	*C. sabaeus*	Vero E6	Custom	Survival	(95)
	SARS-CoV-2	IDF0372	KO	Gattinara	Calu-3	Custom	Survival	(95)
	SARS-CoV-2	IDF0372	a	Calabrese	Calu-3	Custom	Survival	(95)
	SARS-CoV-2	IDF0372	KO	Human ISG CRISPR KO	A549-ACE2	MAGeCK	FACS	(154)
	SARS-CoV-2	nCoV-SH01	KO	Brunello	A549-ACE2	MAGeCK	Survival	(98)
	SARS-CoV-2	SB3-TYAGNC	KO	Toronto Knockout v3	Vero E6, Calu-3, UM-UC-4, HEK-293, Huh-7	Custom	Survival	(136)
	SARS-CoV-2	USA-WA1	KO	GeCKO v2	A549-ACE2	MAGeCK	Survival	(110)
	SARS-CoV-2	USA-WA1	KO	Brunello	Calu-3	MAGeCK	Survival	(50)
	SARS-CoV-2	USA-WA1	a	Calabrese	Calu-3	MAGeCK	Survival	(50)
	SARS-CoV-2	USA-WA1	a	Custom: ISG	A549-ACE2	MAGeCK	Survival	(150)
	SARS-CoV-2	USA-WA1	KO	Custom	Definitive endoderm cells	Custom	Survival	(130)
	SARS-CoV-2	USA-WA1	KO	Custom	Calu-3	MAGeCK	Survival	(158)
	SARS-CoV-2	Pseudotyped USA-WA1	KO	Brunello	HEK-293T-ACE2	Custom	Survival	(97)
	SARS-CoV-2	CLIMVIB2, MIG457, USA-WA1	KO	GeCKO v2	Calu-3	Custom	Survival	(132)
	SARS-CoV-2	UT-NCGM02	KO	Human Improved Genome-wide KO	A549	MAGeCK	Survival	(139)
	SARS-CoV-2	Mouse-adapted QLD02 (MA1)	KO	Brunello	HEK-293T	MAGeCK	Survival	(102)
	SARS-CoV-2	USA-WA1	KO	Brunello, Custom	Huh-7.5	MAGeCK	Survival	(113), (114)
	HCoV-229E	VR-740	KO	Brunello, Custom	Huh-7.5	MAGeCK	Survival	(113), (114)
	HCoV-NL63	0810024CF	KO	Brunello, Custom	Huh-7.5	MAGeCK	Survival	(113), (114)
	HCoV-OC43	N/A	KO	Brunello, Custom	Huh-7.5	MAGeCK	Survival	(113), (114)
	SARS-CoV-2	USA-WA1	KO	GeCKO v2	Huh-7.5.1	MAGeCK	Survival	(100)
	HCoV-229E	VR-740	KO	GeCKO v2	Huh-7.5.1	MAGeCK	Survival	(100)
	HCoV-OC43	VR-1558	KO	GeCKO v2	Huh-7.5.1	MAGeCK	Survival	(100)
	SARS-CoV-2	USA-WA1	KO	*C. sabaeus*	Vero E6	Custom	Survival	(94)
	SARS-CoV-1	HKU5	KO	*C. sabaeus*	Vero E6	Custom	Survival	(94)
	MERS-CoV	NR-48811, NR-48813	KO	*C. sabaeus*	Vero E6	Custom	Survival	(94)
	SARS-CoV-2	UF-1	KO	Brunello, *C. sabaeus*	HEK-293T-ACE2, Vero E6	MAGeCK	Survival	(116)
	HCoV-OC43	N/A	KO	Brunello, *C. sabaeus*	HEK-293T-ACE2, Vero E6	MAGeCK	Survival	(116)
	SARS-CoV-2	Singapore/2	KO	Brunello	IGROV-1	MAGeCK	Survival	(96)
	HCoV-OC43	VR-1558	KO	Brunello	IGROV-1	MAGeCK	Survival	(96)
	SARS-CoV-2	GHB-03021	KO	Brunello	Huh-7	Custom	Survival	(99)
	HCoV-229E	VR-740	KO	Brunello	Huh-7	Custom	Survival	(99)
	TGEV	WH-1	KO	PigGeCKO	PK-15 (porcine)	MAGeCK	Survival	(129)
*Filoviridae*	EBOV	Mayinga	KO	GeCKO v2	Huh-7.5.1	MAGeCK	Survival	(219)
	EBOV	Mayinga; ΔVP30	KO	Brunello	Huh-7.5.1	MAGeCK	FACS	(194)
*Flaviviridae*	DENV	DENV1-276RKI	KO	GeCKO v2	Huh-7.5.1	MAGeCK	Survival	(38)
	DENV	DENV2-429557	KO	GeCKO v2	Huh-7.5.1	MAGeCK	Survival	(38)
	DENV	DENV3-H87185	KO	GeCKO v2	Huh-7.5.1	MAGeCK	Survival	(38)
	DENV	DENV4-BC287/97	KO	GeCKO v2	Huh-7.5.1	MAGeCK	Survival	(38)
	DENV	DENV2-JAM	KO	GeCKO v2	HAP1	MAGeCK	Survival	(69)
	DENV	DENV2-16681	KO	GeCKO v2	Huh-7.5.1	MAGeCK	Survival	(66)
	DENV	DENV2-16681	KO	Custom	Huh-7	MAGeCK	Survival	(85)
	DENV	DENV2-16681	KO	GeCKO v2	Huh-7.5.1	Custom	Survival	(37)
	HCV	JFH1	KO	GeCKO v2	Huh-7.5.1	Custom	Survival	(37)
	JEV	RP9	KO	PigGeCKO	PK-15	MAGeCK	Survival	(159)
	WNV	NY99	KO	Custom	HEK-293T	Custom	Survival	(71)
	WNV	New York 2000	KO	GeCKO v2	HEK-293T	MAGeCK	Survival	(65)
	YFV	17D	KO	Brunello	Huh-7.5 + IFN I treatment	MAGeCK	FACS	(87)
	YFV	17D	KO	Brunello	Huh-7.5	MAGeCK	FACS	(84)
	YFV	Asibi	KO	GeCKO	B3GALT6^-/-^ HAP1	MAGeCK	Survival	(79)
	ZIKV	PRVABC59	KO	GeCKO	B3GALT6^-/-^ HAP1	MAGeCK	Survival	(79)
	ZIKV	PRVABC59	a	SAM v1	STAT1^-/-^ fibroblasts	MAGeCK	Survival	(88)
	ZIKV	MR766	KO	GeCKO v2	Huh-7.5	MAGeCK	Survival	(81)
	ZIKV	MR766	KO	GeCKO v2	H1-HeLa	Custom	Survival	(64)
	ZIKV	MR766	KO	Brunello	Neural progenitor cells	Custom	Survival	(59)
	ZIKV	MR766	KO	Human Activity-Optimized CRISPR KO	Neural progenitors	Custom	Survival	(70)
	ZIKV	MR766	a	SAM v2	Huh-7	MAGeCK	Survival	(86)
	ZIKV	ZsGreen	KO	Brunello	Glioblastoma stem cells	STARS	FACS	(60)
*Hepadnaviridae*	HBV-integrated	Genotype D	KO	Custom	HepG2-HBV	Custom	FACS	(168)
*Hepeviridae*	HEV	83–2	KO	GeCKO v2	Huh-7.5	MAGeCK	Survival	(220)
*Herpesviridae*	EBV	N/A	KO	Brunello	LCL	MAGeCK	FACS	(221)
	EBV	N/A	KO	Avana	BL	STARS	FACS	(222)
	HCMV	TB40E	KO	GeCKO v2	ARPE-19	Custom	Survival	(223)
	HCMV	AD169	KO	GeCKO v2	HEL	Custom	Survival	(223)
	HCMV	AD169	KO	GeCKO v2	Primary human foreskin fibroblasts	MAGeCK	Survival	(224)
	HCMV	GW	KO	Custom	Hs27	Custom	Survival	(192)
	HSV-1	KOS	KO	GeCKO v2	SK-N-SH	MAGeCK	FACS	(225)
	KSHV	N/A	KO	Brunello	iSLK	MAGeCK	FACS	(226)
	KSHV	N/A	KO	GeCKO v2	SLK-iBAC	MAGeCK	FACS	(51)
	MCMV	3DR	KO	Brie	Nrp1^-/-^ 3T3/NIH fibroblasts	Custom	FACS	(177)
	BoHV-1	Jura	KO	Custom	Madin-Darby bovine kidney (MDBK) cells	MAGeCK	FACS	(227)
*Orthomyxoviridae*	IAV	Pseudotyped H18N11	KO	Brunello	U-87MG	MAGeCK	Survival	(228)
	IAV	H5N1	KO	GeCKO v2	A549	Custom	Survival	(229)
	IAV	H5N1	KO	GeCKO v2	A549	Custom	Survival	(40)
	IAV	H7N9	KO	GeCKO v2	A549	MAGeCK	Survival	(48)
	IAV	PR8	KO	Custom	PaKi (bat)	Custom	Survival	(189)
	IAV	PR8	KO	Avana	A549	MAIC	FACS	(41)
	IAV	PR8	KO	Avana	A549 + IFN I treatment	Custom	FACS	(170)
	IAV	PR8	a	Calabrese	HeLa	STARS	Survival	(230)
	IAV	PR8	a	SAM v2	A549	MAGeCK	TRPPC	(191)
	IAV	PR8	a	SAM v2	A549	Custom	FACS	(231)
	IAV	WSN	KO	GeCKO	A549	MAGeCK	Survival	(193)
	IBV	Yam/88	a	Calabrese	A549	MAGeCK	Survival	(47)
*Paramyxoviridae*	SeV	Cantell	KO	Custom	HeLa	Custom	Immunostaining	(232)
*Papillomaviridae*	HPV	18PsV-BE2	KO	GeCKO v2	HeLa	MAGeCK	Survival	(233)
*Parvoviridae*	AAV	AAV9	KO	GeCKO v2	Huh-7	MAGeCK	FACS	(234)
	AAV	rh32.33	KO	GeCKO v2	Huh-7	MAGeCK	FACS	(53)
	AAV	scAAV2	KO	Brunello	HeLa	MAGeCK	Survival	(235)
	Parvovirus	B19	KO	Brunello	UT7/Epo-S1	MAGeCK	FACS	(236)
*Phenuiviridae*	RVFV	ZH501	KO	Brie	BV2	Custom	Survival	(237)
	SFTSV	HB29	KO	GeCKO v2	HEK-293FT	MAGeCK	Survival	(238)
	SFTSV	HBMC16	KO	GeCKO v2	Huh-7	Custom	FACS	(49)
*Picornaviridae*	Enterovirus A	CV-A6	KO	GeCKO v2	A549	MAGeCK	Survival	(239)
	Enterovirus B	CV-B3	KO	GeCKO v2	HeLa	MAGeCK	Survival	(240)
	EMCV	VR-129B	KO	GeCKO v2	H1-HeLa	Custom	Survival	(241)
	HAV	HM175/18f-Tat	KO	Brunello	HeLa-tkGFP	MAGeCK	Survival	(167)
	HAV	HM175/18f	KO	GeCKO v2	Huh-7.5.1	Custom	Survival	(242)
	PeV-A1	Harris	KO	Brunello	HT29-DKO (ΔEXTL3 and ΔSLC35A1)	MAGeCK	Survival	(165)
	PeV-A2	Williamson	KO	Brunello	HT29-DKO (ΔEXTL3 and ΔSLC35A1)	MAGeCK	Survival	(165)
	Enterovirus C	PV1-Chat	KO	Custom	HeLa	Custom	Survival	(243)
	Enterovirus D	EV-D68	KO	Custom	HeLa	Custom	Survival	(243)
	Enterovirus D	EV-D68	KO	GeCKO v2	H1-HeLa	MAGeCK	Survival	(52)
	Rhinovirus C	RV-C15	KO	GeCKO v2	H1-HeLa	MAGeCK	Survival	(52)
	Senecavirus A	SVV	KO	GeCKO v2	HAP1	Custom	Survival	(244)
*Poxviridae*	VV	WR	KO	GeCKO v2	HeLa	MAGeCK	Survival	(245)
*Reoviridae*	Reovirus	T3DCD	KO	GeCKO v2	C57/BL6 embryonic fibroblasts	MAGeCK	Survival	(182)
	RV bovine	NCDV	KO	GeCKO v2	H1-HeLa	MAGeCK	Survival	(246)
*Retroviridae*	HIV	JRCSF	KO	Custom	GXRCas9	Custom	FACS	(45)
	HIV	LAI, Q23.BG505	KO	Custom: CD4-ISG	Primary hCD4+ T Cells	MAGeCK	Survival	(162)
	HIV	LAI	KO	Custom: ISG	THP-1 + IFN I treatment	MAGeCK	Survival	(169)
	HIV	LAI, LAI-VSVG, Q23.BG505, CH470 T/F	KO	Custom	Jurkat	MAGeCK	Survival	(161)
	HIV	Pseudotyped HIV-1	KO	Custom: ISG	U87-MG	Custom	Survival	(163)
	HIV	Pseudotyped HIV-1	KO	GeCKO v2	T98G	MAGeCK	Survival	(247)
	MLV	MSCV	KO	Genome-wide Mouse Lentiviral v2	Murine ESC	MAGeCK	FACS	(183)
	MLV	IN(D184A)	KO	Brunello	HeLa	HiTSelect	FACS	(248)
*Togaviridae*	CHIKV	181/25	KO	GeCKO v2	3T3	MAGeCK	FACS	(35)
	CHIKV	06-21	KO	GeCKO v2	HAP1	MAGeCK	Survival	(34)
	SINV	N/A	KO	Brunello	HCT116	MAGeCK	Survival	(249)
	VEEV	Chimeric TrD	KO	Mouse GeCKO v2	B4galt7^-/-^ Neuro2a	MAGeCK	FACS	(178)
	WEEV	71 V1658	KO	Custom	HEK-293T	MAGeCK	Survival	(160)

^*a*^ a = activation; KO = knockout; N/A = not applicable.
